# A patient‐based dosimetric study of intracavitary and interstitial brachytherapy in advanced stage carcinoma of the cervix

**DOI:** 10.1120/jacmp.v15i3.4509

**Published:** 2014-05-08

**Authors:** Anil K. Bansal, Manoj K. Semwal, Daya N. Sharma, Sanjay Thulkar, Pramod K. Julka, Goura K. Rath

**Affiliations:** ^1^ Department of Radiation Oncology Max Cancer Centre, Max Healthcare New Delhi India; ^2^ Department of Radiotherapy Army Hospital (R&R), Delhi Cantonment New Delhi India; ^3^ Department of Radiotherapy Dr BRA Institute Rotary Cancer Hospital, All India Institute of Medical Sciences New Delhi India; ^4^ Department of Radiodiagnosis Dr BRA Institute Rotary Cancer Hospital, All India Institute of Medical Sciences New Delhi India

**Keywords:** brachytherapy, intracavitary, interstitial, cancer cervix, dosimetry

## Abstract

Intracavitary brachytherapy (ICBT) and interstitial brachytherapy (IB) techniques are commonly practiced for treating carcinoma of the cervix, either alone or in combination with external beam radiotherapy. Both these brachytherapy techniques have their own advantages and limitations in terms of tumor coverage and normal tissue sparing. Limited studies have been reported comparing the dosimetric features of these two techniques, especially from a single institution. We carried out a prospective clinical dosimetric comparison between ICBT and IB for patients treated at one center to bring out the inherent dosimetric features of these to two techniques. The study was carried out on 26 patients treated with ICBT and 55 with IB using CT‐based planning. Of the 55 patients treated with IB, 27 included tandem source loading (IBT) and 28 without the tandem loading (IBWT). The high‐dose volumes covered by 200% and 180% isodose surfaces were considerably larger in ICBT as compared to IB, whereas the treated volume was larger in IB as compared to ICBT. The bladder and rectal doses were the highest in ICBT and IBWT, respectively. The larger treated volume in IB as compared to ICBT was mainly because patients with larger tumor volumes were generally considered for IB. The results also indicated that in interstitial brachytherapy, better rectal sparing was achieved by including the tandem for treatment delivery.

PACS numbers: 87.53.Bn, 87.53.Jw, 87.55.D‐, 87.55.dk

## I. INTRODUCTION

Brachytherapy is a crucial component of radiotherapy in the management of carcinoma of the cervix. In brachytherapy, intracavitary brachytherapy (ICBT) is the most widely used technique for the purpose. However, interstitial brachytherapy (IB) is recommended for cases in which either ICBT is expected to result in a suboptimal dose distribution or it is technically not possible.^1^, ^2^ For IB, the most common approach is the perineal template‐based implant procedure.

Of late, three‐dimensional, image‐based planning with high‐dose‐rate (HDR) treatment delivery system is increasingly being used for both ICBT and IB. The system of point dose prescription and reporting based on orthogonal radiographic reconstruction of applicators/implants (2D planning) has been shown to have limitations as compared to 3D volume dose prescription and reporting.^3^, ^4^ Many studies have found that the 2D planning gives incorrect estimation of dose to organs at risk (OARs).^5^, ^6^, ^7^ To standardize 3D image‐based planning in cervical cancer brachytherapy, guidelines issued by the Image‐Guided Brachytherapy Working Group and the Groupe European de Curethérapie‐European Society for Therapeutic Radiology and Oncology (GEC‐ESTRO) working group have been widely accepted.^8^, ^9^, ^10^ These guidelines also include proposals for research in image‐based brachytherapy for cervical cancer.

A dosimetric comparison between ICBT and IB in cervical cancer brachytherapy is relevant for better appreciation of the clinical outcomes of the two techniques. Limited work is reported in literature on such comparative studies. Hsu et al.^(11)^ carried out one such study based on a hypothetical computer simulation model. A water phantom‐based study involving bladder and rectum dose measurements in ICBT has been reported by Bansal et al.^(12)^ Patient‐based dosimetric comparisons reported in literature are generally retrospective studies related to patients treated at different institutions with ICBT and IB modalities.^13^, ^14^, ^15^ In the present work, we carried out a prospective dosimetric study on two groups of patients planned and treated with Ir‐192‐based HDR ICBT and IB for advanced stage carcinoma cervix at one center by a common team of physicians and physicists. A total of 81 patients were included in this study. The variations due to external factors such as differences in contouring and treatment planning protocols were thus minimized, making the comparison between the two brachytherapy techniques more meaningful. Various parameters such as treated volume, total reference air kerma (TRAK), and doses received by OARs were estimated for the two groups of patients. The present study only compares physical doses without consideration of the radiobiological factors.

## II. MATERIALS AND METHODS

### A. Patients

Consecutive cervical cancer patients (FIGO stage IIIB) treated with brachytherapy following EBRT were included in this study. Eligibility requirements for patient inclusion were: minimum hemoglobin 10 gm, performance status 70 or more (Karnofsky Scale), and histopathological confirmation of the disease. All patients were evaluated for brachytherapy after completion of EBRT. As a policy, patients not found suitable for ICBT were considered for IB. Narrow vagina, parametrial spread of disease, and nonnegotiable passage for tandem were the main reasons for unsuitability of the patients for ICBT. The EBRT protocol followed was 40 Gy in 22 fractions in 4.5 weeks to whole pelvis by four field techniques on a telecobalt or a linear accelerator with 15/18 MV photons. This was followed by 10 Gy in 5 fractions with anterior and posterior fields using a midline shielding block (total 50 Gy in 5.5 weeks). ICBT was delivered in 3 fractions of 7 Gy each and IB in 2 fractions of 9 Gy each. However, for dosimetric comparison, 7 Gy per fraction was considered for both ICBT and IB. A total of 26 patients of ICBT and 55 of IB were included in the study. In the IB group a further distinction was made based on whether a central tandem was used or not for source loading. Of the 55 IB cases, 27 were treated with tandem (IBT) and 28 without the tandem in place (IBWT). Only first fraction of the brachytherapy treatment for each patient was considered in this study.

### B. Intracavitary brachytherapy

Patient's recto‐vaginal bimanual examination and subsequent ICBT applicator placement was done under general anesthesia. First a Foley balloon catheter was inserted into the bladder, and the balloon filled with 7 cc of diluted radio‐opaque contrast solution. The Fletcher Williamson applicator system (Nucletron, Veenendaal, the Netherlands) used for ICBT consisted of an intrauterine tube (tandem) and tilted cylindrical vaginal ovoids. The length of the tandem to be inserted into the uterus was determined with a uterine sound. A lockable flange was placed on the tandem at the sounded distance, and the tandem was then inserted. The flange abutted the external os (EOS), restricting further superior movement of the tandem and thus preventing uterine perforation. The ovoids were positioned within the vaginal vault in the lateral cervical fornices. The largest ovoids allowed by the anatomy were used for better dosimetric results. Ovoids were positioned in such a way that intrauterine tandem bisected the vaginal radiation sources. The tandem and ovoids were immobilized with gauze packing, which also aided in sparing of rectum and bladder. A stitch was placed in the vulva to secure the system in place.

### C. Interstitial brachytherapy

The IB procedure including prior per vaginal examination was performed under epidural anesthesia. The examination was performed to assess the dimensions of the tumor, parametrial and paravaginal tissue involvement, and relationship of the tumor to the uterus and other pelvic organs. The Martinez universal perineal interstitial template (MUPIT) was used for needle implantation. After placement of Foley balloon catheter, the tandem was inserted similar to the process of ICBT. The obturator was then moved over the tandem till its ring touched the EOS and locked with a screw at the outer end of the obturator. A guide needle was then inserted mostly through the posterior vaginal wall under the transrectal ultrasound guidance. The tip of this needle was taken 1‐2 cm beyond the clinically palpable disease. Vaginal length was determined and vaginal obturator along with tandem was fixed to the template at that length. The MUPIT was secured to the perineum by means of stitches taken through its peripheral holes. The remaining needles were placed around the vaginal cylinders up to a preset depth. The number and position of needles were decided as per the extent of the disease. The needles were bilaterally symmetrical in most cases, and their number ranged from 18 to 26 to encompass the target volume. Per rectal examination was then carried out to ensure that no needle had pierced the rectal mucosa.

### D. Treatment planning and delivery

Contiguous CT images of 2.5 mm slice thickness from the level of the obturator foramen to 2.0 cm beyond the tandem tip/superior‐most needle tip were acquired on Volume Zoom scanner (Siemens, Erlangen, Germany). The images were exported to Plato brachytherapy treatment planning system version 14.3.7 (Nucletron) through a DICOM network. External contours for bladder, rectum, and sigmoid colon were drawn on the CT axial images. The delineation of the internal wall surface of these organs was generally not possible on CT and hence was not attempted. The position of the recto‐sigmoid junction was defined at the level of the top of two femoral heads, rather than the more commonly used definition in which the junction occurs where the sigmoid colon curves off from the rectum. We used this definition simply for better consistency in delineation due to the bony landmarks, as also reported by Kim et al.^(16)^ Similarly, the rectum was defined as the bowel below the level of the top of the femoral head to the bottom of the coccyx. The sigmoid colon was defined as the bowel above the level of the top of the femoral heads to the level of the lumbosacral interspace. The contouring of the OARs for all patients was done by one experienced person for maintaining the necessary consistency. In the case of ICBT, the applicators (tandem and ovoids) were also contoured for estimation of high‐dose volumes after removing the applicator volume from the DVH.

The position of the EOS on the CT image was defined by the cervical marker ring placed on the tandem. Point A was found relative to the EOS marker, first by measuring 2 cm superiorly along the tandem, and from that point moving 2 cm perpendicular to the tandem in the lateral direction. For ICBT, active dwell positions were kept at 1, 3, 5, 7, 10, 13, and 16 from the tip for uterine tandem, and 1 to 6 (continuous four dwell positions) for the colpostats with 2.5 mm step size in such a way that these positions were bisected by the uterine tandem. In IB, the needles were loaded 4 cm towards the tip and 2 cm towards the open end (total 6 cm length) from the EOS. The step‐size for all dwell positions in IB was 5 mm, except at the first and last positions where it was 2.5 mm. For ICBT, the dose (7 Gy) was prescribed at point A, and for IB it was prescribed at an isodose surface that covered the peripheral needles with a margin of about 5 mm on all sides. This isodose was also called as reference isodose and normalized to 100%.

Cumulative dose‐volume histograms (DVH) were used to estimate treatment volumes covered by 200%, 180%, 150%, 100%, 75%, and 50% isodose surface (V200, V180, V150, V100, V75, and V50, respectively), and minimum dose to 0.1 cc, 1 cc, 2 cc most irradiated volumes ($D_{0.1\text{cc}},D_{1\text{cc}},D_{2\text{cc}}$, respectively) of bladder, rectum, and sigmoid colon. The dose and volume parameters estimated included data recommended by gynecological GEC‐ESTRO working group.^(10)^ Maximum width, length, and height of the prescription isodose surface and TRAK values were also recorded. The treatment was delivered on a microSelectron HDR brachytherapy machine (Nucletron).

### E. Statistical methods

One way analysis of variance (ANOVA) for independent groups was used for analysis. WINKS SDA (version 6.0.9) with Newman‐Keuls multiple comparison method was used for testing the significance at 0.05 level.

## III. RESULTS

Table 1 shows the volumes covered by the various isodose surfaces, dimensions of the volume covered by the prescription isodose surface (V100), and TRAK values for the three categories of implants. The mean V200 and V180 values (high‐dose volumes) for ICBT were significantly higher as compared to that of IB (both with and without tandem). The table also shows that high‐dose volumes in the case of ICBT after removing the applicator volumes covered by the 200% and 180% isodose surfaces (V200‐AV, V180‐AV) continue to be considerably higher as compared to IB. While estimating the high‐dose volumes without applicator volumes from the DVHs, it was found out that mainly ovoids contributed to the applicator volumes, as compared to tandem and needles. Therefore, V200‐AV and V180‐AV values only for ICBT are shown in the table. The V150 value for all the three implant categories was close to one another. The treated volume (V100) was significantly higher for both categories of IB implants, as compared to ICBT. As expected, the treated volume length is not much different among all categories of implants, whereas the width of the implant is larger for IB implants as compared to ICBT implants. The significantly higher treated volume thickness for ICBT as compared to IB could adversely impact the doses received by the OARs, namely bladder and rectum. TRAK values (at one meter) were higher for IB as compared to ICBT, but the difference was statistically not significant.

**Table 1:** Treatment volumes (cc), TRAK, and dimensions of treated volume (V100). Values shown are mean mean±standard deviation (range)

	*IBT*	*IBWT*	*ICBT*
V200	18.0±4.0	18.9±5.5	35.5±1.1
	(10.9 to 25.2)	(11.5 to 29.7)	(30.9 to 40.0)
V200‐AV	–	–	29.32±1.3
			(22.8 to 35.10)
V180	29.1±6.7	29.6±7.8	42.2±0.8
	(17.2 to 37.9)	(18.6 to 44.0)	(36.3 to 47.0)
V180‐AV	–	–	35.5±1.1
			(30.3 to 39.8)
V150	60.3±12.9	57.9±11.5	56.6±0.6
	(35.7 to 80.0)	(40.1 to 80.5)	(20.5 to 38.5)
V100	140.0±13.7	137.3±15	105.2±0.6
	(110.6 to 159.3)	(117.0 to 176.6)	(86.1 to 117.9)
V75	204.1±18.9	202.4±20.8	160.8±1.4
	(162.7 to 231.5)	(168.0 to 254.3)	(130.3 to 180.3)
V50	345.0±32.1	343.0±34.6	288.6±3.8
	(275.5 to 392.5)	(280.5 to 420.6)	(234.0 to 323.0)
*TRAK (mGy at 1 meter)*			
	5.2±0.4	5.20±0.4	5.1±0.1
	(4.5 to 5.8)	(4.4 to 6.0)	(3.4 to 5.5)
*V100 (dimensions in mm)*			
Height	68.8±0.9	68.9±1.1	72.8±6.9
	(67.1 to 70.1)	(66.3 to 70.6)	(62.3 to 79.3)
Width	81.6±4.5	80.9±6.9	62.4±8.3
	(73.0 to 89.7)	(61.8 to 90.3)	(55.1 to 74.5)
Thickness	35.3±2.3	35.3±2.8	44.5±0.3
	(28.8 to 38.6)	(28.9 to 41.9)	(41.4 to 47.3)

TRAK=total reference air kerma (mGy at 1 meter); IBT=interstitial brachytherapy with tandem; IBWT=interstitial brachytherapy without tandem; ICBT=intracavitary brachytherapy; V200,V180…V50=volumes covered by200, 180…50 percent isodose surfaces; V200‐AV, V180−AV=volumes covered by 200 and 180 percent isodose surfaces after removing the applicator volume covered by these isodose surfaces.

Table 2 shows dose estimated for various volumes of bladder and Foley balloon, and absolute bladder volume as drawn on CT images. The bladder dose was the highest in ICBT followed by IBT and IBWT for all the volumes of the bladder considered, though the difference decreased for higher volumes. Statistically analyzing these results revealed that the difference between IB (with or without tandem) and ICBT values was statistically significant. It was also evident from the data presented in the table that the Foley balloon underestimated dose to bladder for all three categories of implants for the corresponding volumes.

**Table 2:** Bladder and Foley balloon dose (cGy). Values shown are mean ± standard deviation (range)

	*IBT*	*IBWT*	*ICBT*
*Bladder*			
$D_{0.1\text{cc}}$	724.9±109.8	698.4±99	1191.3±312.5
	(483.1 to 934.0)	(533.2 to 930.3)	(601.4 to 1795.1)
$D_{1.0\text{cc}}$	632.2±93.4	596.1±82.2	929.7±314
	(428.4 to 831.0)	(465.0 to 757.1)	(487.3 to 1298.4)
$D_{2.0\text{cc}}$	593.2±85.5	554.6±94.7	836.8±282.1
	(403.4 to 748.6)	(423.3 to 690.0)	(446.1 to 1161.2)
Volume	129.4±76.6	159.4±90.9	135.3±36.6
	(38.1 to 385.0)	(40.8 to 371.3)	(36.6 to 362.4)
*Foley Balloon*			
$D_{0.1\text{cc}}$	569.9±151.5	578.6±134.7	616.2±195.2
	(122.7 to 848.8)	(416.5 to 912.6)	(315.0 to 1137.9)
$D_{1.0\text{cc}}$	459.5±114.9	467.6±97.4	485.5±153.4
	(106.1 to 663.0)	(347.8 to 691.1)	(269.0 to 788.3)
$D_{2.0\text{cc}}$	404.9±98.5	411.5±80	422.2±135.8
	(98.1 to 576.2)	(310.1 to 587.4)	(242.9 to 648.3)

IBT=interstitial brachytherapy with tandem; IBWT=interstitial brachytherapy without tandem; ICBT=intracavitary brachytherapy; $D_{0.1\text{cc}},D_{1.0\text{cc}},D_{2.0\text{cc}}=$ minimum dose received by most irradiated volumes 0.1 cc, 1.0 cc, 2.0 cc.

Rectum and sigmoid doses are shown in Table 3. For the range of rectal volumes considered (0.1 cc to 2 cc), the dose was estimated to be higher for IBWT, as compared to ICBT and IBT. The difference was statistically significant between IBT and IBWT, as well as between ICBT and IBWT, but not between IBT and ICBT. Also to be noted was the larger standard deviation in the dose values for ICBT, as compared to IB. In the case of sigmoid, the mean dose values did not differ much between the three implant techniques. Though it can be said that similar to the rectum dose pattern, the maximum (mean) dose in sigmoid was for IBWT for volumes 1.0 cc and 2.0 cc.

**Table 3:** Rectum and sigmoid dose (cGy) and volumes (cc). Values shown are mean ± standard deviation (range)

	*IBT*	*IBWT*	*ICBT*
*Rectum*			
$D_{0.1\text{cc}}$	706±78.4	802.9±119.7	778.9±700.7
	(562.3 to 878.6)	(579.0 to 1065.3)	(298.2 to 1298.1)
$D_{1.0\text{cc}}$	597.7±76.4	663.6±98.8	609.8±484.4
	(441.9 to 790.2)	(461.5 to 838.3)	(270.0 to 955.0)
$D_{2.0\text{cc}}$	545.2±74.7	606.3±94.7	538.6±382.5
	(392.1 to 741.4)	(398.7 to 753.1)	(257.5 to 797.1)
Volume	23.7±8.4	24.2±12.7	28.2±1.3
	(12.0 to 47.1)	(12.2 to 56.2)	(15.7 to 63.3)
*Sigmoid*			
$D_{0.1\text{cc}}$	469±180	498.1±184.5	504.6±18.4
	(221.2 to 851.0)	(192.0 to 921.1)	(252.9 to 772.0)
$D_{1.0\text{cc}}$	373±135.9	392.3±134.5	380.3±12.7
	(178.0 to 686.3)	(164.5 to 704.5)	(210.3 to 553.1)
$D_{2.0\text{cc}}$	335±119.6	348.4±115.2	332.8±3.5
	(158.6 to 603.2)	(151.0 to 606.1)	(187.3 to 496.9)
Volume	24.1±9.7	27.4±12.4	28±1.8
	(10.2 to 59.1)	(10.8 to 53.0)	(10.1 to 71.8)

IBT=interstitial brachytherapy with tandem, IBWT=interstitial brachytherapy without tandem; ICBT=intracavitary brachytherapy; $D_{0.1\text{cc}},D_{1.0\text{cc}},D_{2.0\text{cc}}=\text{minimum dose received by most irradiated}$ volumes 0.1 cc, 1.0 cc, 2.0 cc.

## IV. DISCUSSION

The results of this study show that the treated volume is larger with IB, as compared to ICBT, with similar or better sparing of the OARs. Further, the high‐dose volumes (V200, V200‐AV V180, V180‐AV) in the case of ICBT are considerably larger as compared to IB, but may not be always undesirable in brachytherapy. V200, V150, V100, and V50 values reported by Fellner et al.^(5)^ with a ring applicator system were 31±1,51±3,98±9, and 275±30cc, respectively. These values are close to the corresponding values estimated in the present study (i.e., 35.5±1.1,56.6±0.6,105.2±0.5, and 288.6±3.8cc, respectively). In a computer model‐based study by Hsu et al.,^(11)^ the V180 values were 31 cc and 17 cc for ICBT and IB, as compared to our corresponding values of 42.2 and 29.1 cc, showing similar volume pattern between ICBT and IB. The larger treated volume in IB is mainly a result of the patient selection policy at our center wherein patients with bulkier tumors generally undergo IB. The larger average width of the treatment volume V100 in IB, as shown in Table 1, means better coverage of parametrial regions in IB. The median width of reference isodose as 73 mm and 63 mm for IB and ICBT, respectively, reported by Saha et al.,^(13)^ is close to values reported by us in terms of difference between IB and ICBT.

The bladder dose was higher in ICBT as compared to IB, whereas the rectum and sigmoid doses were in the similar ranges in both the implant systems. The higher bladder dose in ICBT as compared to IB can be attributed to the steeper dose falloff outside the prescription isodose surface in IB as compared to ICBT. In IB, the radioactive source is closer to the prescription isodose surface as compared to ICBT and, hence, there is a steeper dose falloff in the close vicinity of the source mainly governed by the inverse square law. Better sparing of bladder and rectum in IB has also been reported in literature.^11^, ^13^ The underestimation of bladder dose by the Foley balloon could be attributed to the closeness of lateral pouch of the bladder to the applicator, as compared to the balloon which generally remains at mid plane in the bladder neck. Studies by other groups have also found out that ICRU bladder point generally underestimates the bladder maximum dose.^5^, ^16^, ^17^, ^18^

The lower dose to rectum in IBT and ICBT as compared to IBWT could be due to the presence of the tandem that generally pulled the isodoses anteriority due to antiflexion of uterus. There is a larger patient‐to‐patient variation in the rectum, sigmoid, and bladder dose values for ICBT, as indicated by the larger standard deviation values. This could be due to higher dependence of implant geometry on the patient anatomy in ICBT as compared to IB. Rutten et al.^(19)^ have also pointed to this kind of variation in ICBT due to the differences in applicator placement. Kim et al.^(20)^ in their study of 13 patients of cancer cervix treated by ICBT using radiographic film planning reported the mean bladder, rectum, and sigmoid colon $D_{2\text{cc}}$ values as 632.6 cGy, 501.1 cGy, and 531.5 cGy, respectively, for a point A prescription dose of 800 cGy. The corresponding values in our ICBT study are 836.8 cGy, 538.6 cGy, and 332.8 cGy, respectively, for a prescription dose of 700 cGy to point A. A higher $D_{2\text{cc}}$ value for bladder in our case as compared to the quoted study could be due to a higher degree of antiflexion of uterus for most of the patients in the present study, and a possible difference in the procedure followed for effective vaginal packing.

## V. CONCLUSIONS

A decrease in bladder dose and increase in rectal dose in IBWT as compared to IBT implies that, if rectal toxicity is an overriding concern, IBT should be attempted wherever feasible. In the present work, target volumes were not drawn for planning and hence parameters, such as D100‐, and D90 (minimum doses received by 100% and 90% of the target volumes) recommended by GEC‐ESTRO guidelines, could not be estimated. Further, though the volumetric physical dose comparison presented in this study is superior to point‐dose estimation, yet incorporation of radiobiological parameters may change the nature of this comparison and the conclusions drawn. Further study incorporating the radiobiological parameters is being considered for analyzing their clinical impact.

## References

[1] Nag S , Erickson B , Thomadsen B , Orton C , Demanes JD , Petereit D . The American Brachytherapy Society recommendations for high‐dose‐rate brachytherapy for carcinoma of the cervix. Int J Radiat Oncol Biol Phys. 2000;48(1):201–11. 10924990 10.1016/s0360-3016(00)00497-1

[2] Kuipers T , Hoekstra CJM , Van Riet A , et al. HDR brachytherapy applied to cervical carcinoma with moderate lateral expansion: modified principles of treatment. Radiother Oncol. 2001;58(1):25–30. 11165678 10.1016/s0167-8140(00)00320-0

[3] International Commission on Radiation Units and Measurements . ICRU Report 38: Dose and volume specification for reporting intracavitary therapy in gynecology. Bethesda, MD: ICRU; 1985.

[4] International Commission on Radiation Units and Measurements . ICRU Report 58: Dose and volume specification for reporting interstitial therapy. Bethesda, MD: ICRU; 1997.

[5] Fellner C , Potter R , Knocke TH , Wambersie A . Comparison of radiography‐ and computed tomography‐based treatment planning in cervix cancer in brachytherapy with specific attention to some quality assurance aspects. Radiother Oncol. 2001;58(1):53–62. 11165682 10.1016/s0167-8140(00)00282-6

[6] Wachter‐Gerstner N , Wachter S , Reinstadler E , et al. Bladder and rectum dose defined from MRI based treatment planning for cervix cancer brachytherapy: comparison of dose‐volume histograms for organ contours and organ wall, comparison with ICRU rectum and bladder reference points. Radiother Oncol. 2003;68(3):269–76. 13129634 10.1016/s0167-8140(03)00189-0

[7] Jemema SV , Saju S , Mahantshetty U , et al. Dosimetric evaluation of rectum and bladder using image‐based CT planning and orthogonal radiographs with ICRU 38 recommendations in intracavitary brachytherapy. J Med Phys. 2008;33(1):3–8. 20041045 10.4103/0971-6203.39417 PMC2786096

[8] Nag S , Cardenes H , Chang S , et al. Proposed guidelines for image‐based intracavitary brachytherapy for cervical carcinoma: report From Image‐Guided Brachytherapy Working Group. Int J Radiat Oncol Biol Phys. 2004;60(4):1160–72. 15519788 10.1016/j.ijrobp.2004.04.032

[9] Haie‐Meder C , Potter R , Limbergen EV , et al. Recommendations from Gynaecological (GYN) GEC ESTRO working group (I): concepts and terms in 3D image based 3D treatment planning in cervix cancer brachytherapy with emphasis on MRI assessment of GTV and CTV. Radiother Oncol. 2005;74(3):235–45. 15763303 10.1016/j.radonc.2004.12.015

[10] Potter R , Haie‐Meder C , Limbergen EV , et al. Recommendations from Gynaecological (GYN) GEC ESTRO working group (II): concepts and terms in 3D image‐based treatment planning in cervix cancer brachytherapy—3D dose volume parameters and aspects of 3D image‐based anatomy, radiation physics, radiobiology. Radiother Oncol. 2006;78(1):67–77. 16403584 10.1016/j.radonc.2005.11.014

[11] Hsu IJ , Speight J , Hai J , Vigneault E , Phillips T , Pouliot J . A comparison between tandem and ovoids and interstitial gynecologic template brachytherapy dosimetry using a hypothetical computer model. Int J Radiat Oncol Biol Phys. 2002;52(2):538–43. 11872302 10.1016/s0360-3016(01)02691-8

[12] Bansal AK , Semwal MK , Arora D , Sharma DN , Julka PK , Rath GK . A phantom study on bladder and rectum dose measurements in brachytherapy of cervix cancer using FBX aqueous chemical dosimeter. Phys Med. 2013;29(4):368–73. 22687710 10.1016/j.ejmp.2012.05.005

[13] Saha A , Dastidar AG , Sau S , Mallik S , Jayanti J , Basu A . 3D image‐guided HDR brachytherapy in locally advanced cervix cancer ‐ comparison of intracavitary and interstitial treatment [abstract]. Brachytherapy. 2007;6(2):77–78.

[14] Monk BJ , Tewari K , Burger RA , Johnson MT , Montz FJ , Berman ML . A comparison of intracavitary versus interstitial irradiation in the treatment of cervical cancer. Gynecol Oncol. 1997;67(3):241–47. 9441770 10.1006/gyno.1997.4877

[15] Saitoh J , Ohno T , Sakurai H , et al. High‐dose‐rate interstitial brachytherapy with computed tomography‐based treatment planning for patients with locally advanced uterine cervical carcinoma. J Radiat Res. 2011;52(4):490–95. 21785237 10.1269/jrr.10189

[16] Kim RY , Shen S , Duan J . Image‐based three‐dimensional treatment planning of intracavitary brachytherapy for cancer of the cervix: dose‐volume histograms of the bladder, rectum, sigmoid colon, and small bowel. Brachytherapy. 2007;6(3):187–94. 17606413 10.1016/j.brachy.2006.11.005

[17] Tan LT , Warren J , Freestone G , Jones B . Bladder dose estimation during intracavitary brachytherapy for carcinoma of the cervix using a line source system. Br J Radiol. 1996;69(826):953–62. 9038532 10.1259/0007-1285-69-826-953

[18] Addeo D , Duckworth T , Blank S , Hitchen C , Donach M , Formenti S . Correlation between (ICRU) point doses and CT based image planning of intracavitary brachytherapy for cervical cancer [abstract]. Proceedings of the 50th Annual ASTRO Meeting. Int J Radiat Oncol Biol Phys. 2008;72(1):S374.

[19] Rutten RR , Lawyer AA , Berner P . Dose variation due to differences in applicator placement used for intracavitary brachytherapy of cervical cancer. Med Dosim. 1998;23(1):57–63. 9586723 10.1016/s0958-3947(97)00110-6

[20] Kim R , Shen S , De Los Santos J , Spencer S . Factors affecting ICRU point dose and 3‐D volume dose for organs at risk in image‐based intracavitary brachytherapy planning for cervical cancer [abstract]. Proceedings of the 50th Annual ASTRO Meeting. Int J Radiat Oncol Biol Phys. 2008;72(Suppl):S373–374.

